# A multi-purpose dataset of Devanagari script comprising of isolated numerals and vowels

**DOI:** 10.1016/j.dib.2021.107723

**Published:** 2021-12-16

**Authors:** Duddela Sai Prashanth, R Vasanth Kumar Mehta, Nagendra Panini Challa

**Affiliations:** a SCSVMV Univesity, Kanchipuram 631561, India; b Sahyadri College of Engineering and Management, Mangaluru 575005, India; c Shri Vishnu Engineering College for Women (A), Bhimavaram 534202, India

**Keywords:** Devanagari dataset, Handwritten character, Computer vision, Machine learning

## Abstract

This article presents handwritten isolated characters of the Devanagari script. Devanagari script contains ten numerals, 13 vowels, and 33 consonants. Devanagari Character dataset includes 23 different characters of numerals and vowels. 2400 handwritten samples are collected for each of the numerals and 1400 for each vowel. Collected samples are digitized and pre-processed. During pre-processing, images with noise are removed. In this context, a final dataset of 38,750 images were included, where 2,250 and 1,250 samples for each numeral and vowel, respectively. The data is available in images and comma-separated-values, along with attached labels. The dataset could be used for Optical Character Recognition research and deep learning. In India, the Devanagari script is the base script on which 120+ languages are evolved; hence this dataset serves as the base for Machine Learning research in these languages. The data set is publicly available at https://data.mendeley.com/datasets/pxrnvp4yy8/2.

## Specifications Table

**Table:** 

Subject	Computer Vision and Pattern Recognition
Specific subject area	Computer Vision, Optical Character Recognition, Machine Learning,
Type of data	Table: comma-separated values, Image: JPEG
How data were acquired	Targeted subjects are from schools and colleges of age groups from 10 to 50. They were requested to write the Devanagari Numerals and Vowels on the plain A4 sheets. Those sheets were scanned using Epson – 150 to scan pages
Data format	Raw, JPEG
Parameters for data collection	A4-sized sheets were distributed to the subjects. The subjects wrote numerals and Vowels in Devanagari Script on the A4 sheet.
Description of data collection	For the preparation of the dataset: • We used the bound box technique to extract the characters from the scanned images. • Removed all the images which contained fewer than 10 pixels. • Converted all the images into grayscale and then to black & white, which helps to reduce the computational cost while developing Machine Learning Models. • We extracted 38,750 sub-images with a size of 28 × 28, which contains Devanagari numerals and vowels. • We generated a comma-separated values format for all the images.
Data source location	Mangaluru, Karnataka India Tirupati, Andhra Pradesh, India
Data accessibility	Repository Name: Mendeley Data doi: 10.17632/pxrnvp4yy8.3 https://data.mendeley.com/datasets/pxrnvp4yy8/3

## Value of the Data

• Devanagari characters were collected from the subjects of different age groups, then pre-processed, resized, and attached labels, which is helpful in developing machine learning models.
• As of date, there are very few data sets available in the Devanagari script (Majid and Smith, 2018) [1]. This contribution provides one of the most extensive handwritten datasets in the Devanagari script, which can be used for building and optimizing machine learning models in computer vision (Liu, 2017) [3].
• Other researchers can use this data as a benchmark dataset in handwritten optical character recognition of Devanagari numerals and vowels.
• The dataset contains 38,750 isolated Devanagari characters composing of 22,500 numerals and 16,250 vowels. The quantity of samples makes it appropriate for deep learning research.
• Due to the lack of a benchmark dataset, unlike MNIST for Latin numbers, the research is limited in Devanagari numerals; hence this data will fill the data gap.
• Many Indian languages include Marathi, Pali, Sanskrit, Hindi, Nepali, Bhojpuri, Haryanvi, Nagpuri, Kashmiri, Konkani, Sindhi, Bodo, Nepalbhasa, to name a few, are derivatives of Devanagari Script (Devanagari, 2020) [4]. Hence, this dataset shall serve as a starting point for Machine Learning Research in these languages.

## 1 Data Description

**Fig. 1 and Fig. 2:**
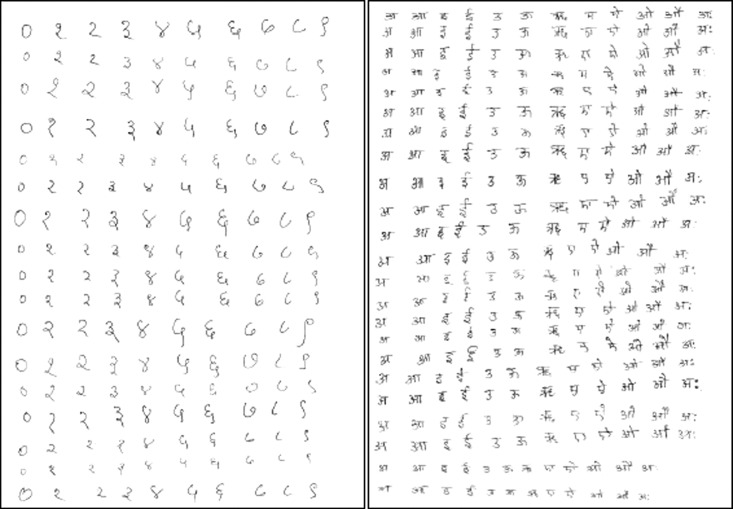
Sample A4 sheets used to collect data.

**Fig. 3:**
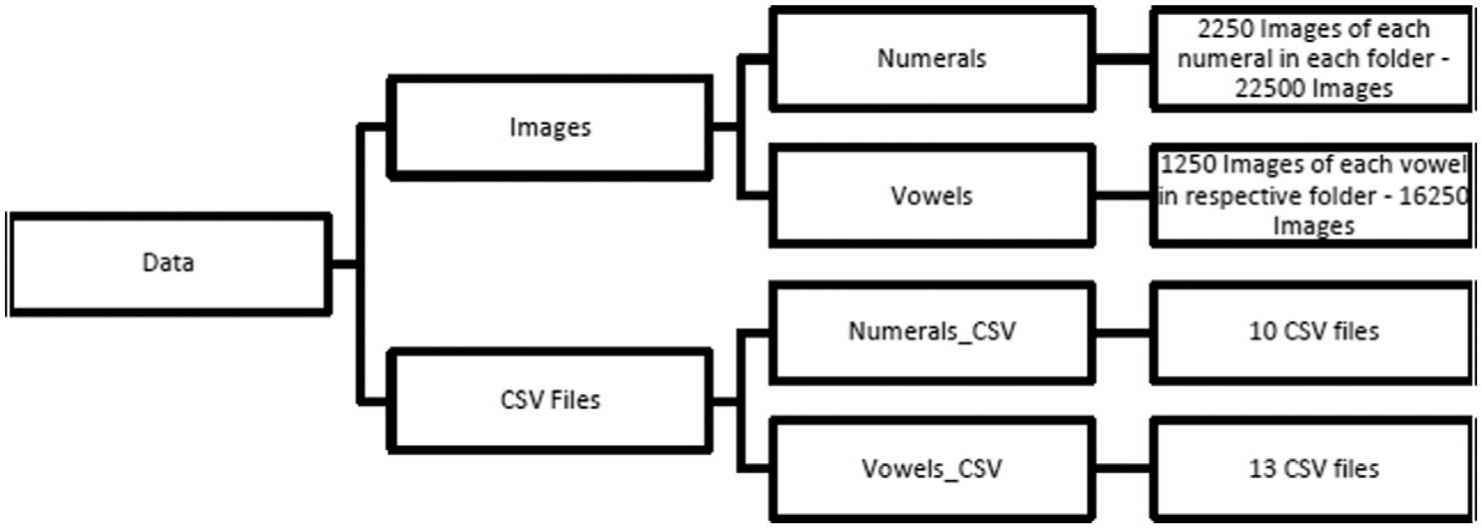
Data is arranged in the zip files.

The data contains handwritten samples of Devanagari numerals and vowels (i.e., 10 numerals and 13 vowels). Thus, the dataset includes 23 different Devanagari characters, as shown in Tables 1 and 2. The data is collected on a regular A4 sheet, scanned at 300 dpi using Epson DS – 150 is shown in Figs. 1 and 2. The numerals and vowels were collected from 2400 and 1400 subjects of different age groups. Further, data is segmented, pre-processed, and stored in a publicly accessible location. By removing the occluded images and scribbles, the final data set contains a total of 38,750 digitized images where 22,500 Devanagari Numerals (2250 each) and 16,250 Vowels (1250 each). This data was separated manually into respective folders, as shown in Fig. 3. A total number of 23 CSV files are in the dataset. Each CSV file represents a different character or numerals. Each directory of the images represents the respective type.

**Table 1:** Devanagari numerals representation.

Numerals	0	1	2	3	4	5	6	7	8	9
Numerals Sample Image										
										
Class Number	10	1	2	3	4	5	6	7	8	9

**Table 2:** Devanagari vowels representation.

Numerals	a	aa	e	ee	u	uu	ru	ye	i	o	au	am	aha
Numerals Sample Image													
													
Class Number	11	12	13	14	15	16	17	18	19	20	21	22	23

## 2 Experimental Design, Materials and Methods

### 2.1 Data collection

The success rate of research on the recognition of handwritten English characters is high compared to the Indian script like Devanagari. The state-of-the-art techniques in deep learning are efficient in automatically recognizing Devanagari handwritten characters, but this requires large data samples with labels. This data will fill that data gap for Devanagari numerals and vowels [2].

The subjects were asked to write isolated Devanagari numbers and vowels on a plain A4 sheet, as shown in Figure 1 and Fig. 2. The data is collected from the subjects of different age groups of 10 – 50, which helps get different data samples.

### 2.2 Data processing

All the forms are scanned using the Epson scanner at 300 dpi and stored in JPEG format. Characters are extracted from the scanned images using the bound box technique. The segmented characters are manually segregated since noise in the scanned images is also obtained as characters. All the images were resized to 28 × 28 pixels and verified manually. The vision for creating this data is to make better Machine Learning models, and the extracted images were converted into black and white. Where background was converted to black and character was converted to white, as shown in Tables 1 and 2.

Extracted images of Devanagari characters were arranged into different folders—a total of 23 folders for each character. The names of the folders and which image files were placed are shown in Figure 3.

Every image is converted into an image vector, and a label is attached to it. An image of size 28 × 28 generates a vector of 1 × 784 plus 1 more value, which indicates its label. A total of 23 CSV files were generated, where each file represented each character. Each CSV file of numerals contains 2250 rows for numeral character and 1250 rows for vowels, in which each row represents one image, and the last value represents the label.

## Ethics Statement

All the handwriting characters were obtained with consent from respondents school authority prior to collection of data. Ethical approval was not needed since there was no research involving human subjects or animals.

## CRediT authorship contribution statement

**Duddela Sai Prashanth:** Conceptualization, Methodology, Writing – original draft. **R Vasanth Kumar Mehta:** Supervision. **Nagendra Panini Challa:** Writing – review & editing, Visualization.

## Declaration of Interest

The authors declare that they have no known competing financial interests or personal relationships which have, or could be perceived to have, influenced the work reported in this article.
